# Small Molecule Wnt Pathway Modulators from Natural Sources: History, State of the Art and Perspectives

**DOI:** 10.3390/cells9030589

**Published:** 2020-03-02

**Authors:** Artem Blagodatski, Antonina Klimenko, Lee Jia, Vladimir L. Katanaev

**Affiliations:** 1 School of Biomedicine, Far Eastern Federal University, Vladivostok 690090, Russia; klimenkot.vl@gmail.com; 2 Moscow Institute of Physics and Technology, Dolgoprudny 141701, Russia; 3 Institute of Oceanography, Minjiang University, Fuzhou 350108, China; leejia2000@mju.edu.cn; 4 Fujian Provincial Key Laboratory of Cancer Metastasis Chemoprevention and Chemotherapy, Fuzhou University, Fuzhou 350108, China; 5 Translational Research Center in Oncohaematology, Department of Cell Physiology and Metabolism, Faculty of Medicine, University of Geneva, 1211 Geneva, Switzerland

**Keywords:** natural products, Wnt signaling, cancer, regeneration, drug discover

## Abstract

The Wnt signaling is one of the major pathways known to regulate embryonic development, tissue renewal and regeneration in multicellular organisms. Dysregulations of the pathway are a common cause of several types of cancer and other diseases, such as osteoporosis and rheumatoid arthritis. This makes Wnt signaling an important therapeutic target. Small molecule activators and inhibitors of signaling pathways are important biomedical tools which allow one to harness signaling processes in the organism for therapeutic purposes in affordable and specific ways. Natural products are a well known source of biologically active small molecules with therapeutic potential. In this article, we provide an up-to-date overview of existing small molecule modulators of the Wnt pathway derived from natural products. In the first part of the review, we focus on Wnt pathway activators, which can be used for regenerative therapy in various tissues such as skin, bone, cartilage and the nervous system. The second part describes inhibitors of the pathway, which are desired agents for targeted therapies against different cancers. In each part, we pay specific attention to the mechanisms of action of the natural products, to the models on which they were investigated, and to the potential of different taxa to yield bioactive molecules capable of regulating the Wnt signaling.

## 1. Introduction

### 1.1. Drugs from Natural Sources—A Brief Overview

Drug discovery from natural products (NP) is passing through a renaissance, for the natural treasury of biologically active compounds, which previously gave birth to such essential drugs as penicillin [1], taxol [2], vinca alkaloids [3] and many others, is now entering the scope of targeted and personalized medicine. Cytotoxic compounds and broad-spectrum antibiotics are still being discovered from natural sources and continue to yield Nobel prizes, like the recent ones for the discovery of artemisinin as an anti-malaria agent [4] and avermectin, a novel antibiotic from soil microorganisms [5]. However, compounds with a capacity to specifically interfere with signaling processes of the organism, switching them from a pathological route back to the normal function without harming the uninvolved housekeeping processes, become even more attractive. The majority of the already discovered—and yet to be discovered—NP are still waiting to be examined for their possible role to serve as such tools. There are several approaches to identify molecules with new biologically active properties, such as in silico screening [6], rational design [7] or large-scale screening of libraries of artificially synthetized compounds [8,9]. When comparing these approaches, NP collections appear to have an important advantage over the purely synthetic compounds. Indeed, secondary metabolites have evolved to interact with various protein targets in their plant, fungal, etc. hosts. Given the limited number of possible protein folds, it is conceivable that upon rencontre with human disease proteins, such NP will have a higher chance of finding their match than randomly synthesized compounds would do [10,11,12,13]. Indirect support for this reasoning is provided by the mere fact that ca. 50% of all currently approved drugs are NP-based [14,15].

Speaking of the sources of NP as drug candidates, it is clear that plants, fungi and bacteria dominate. Marine hydrobiome is lagging behind in such exploration but is doomed to catch up, given the high (and perhaps higher) and structurally different chemodiversity it hides as compared with terrestrial organisms [13,16].

### 1.2. Wnt Pathway and Its Biomedical Importance

There are a number of signaling pathways, like Notch [17], TGFβ [18] or Hedgehog [19], that govern the embryonic development of multicellular organisms, later reducing their activity to the fine-tuning of certain biological processes like stem cell renewal or regeneration. Dysregulation of these signaling pathways in adults may lead to diseases such as cancer. The Wnt signaling cascade belongs to this group of embryonic pathways that additionally plays important functions in adulthood, which must be well regulated in order to avoid pathological complications. These main functions of the Wnt pathway are: maintenance of cell proliferation, stem cell self-renewal, tissue regeneration, and neuritogenesis and synaptogenesis in the nervous system [20].

The key signal intermediate in the canonical Wnt signaling is β-catenin, which upon continuous synthesis is degraded in the cytoplasm by a multiprotein destruction complex consisting of scaffolding proteins Axin and APC and two kinases: glycogen synthase kinase 3β (GSK3β) and casein kinase 1 (CK1). This complex traps and phosphorylates β-catenin, thereby targeting it for ubiquitinoylation and proteasomal degradation [21]. With low levels of cytoplasmic and nuclear β-catenin, the canonical Wnt signaling is inactive.

The physiological activators of the pathway are the lypoglycoproteins of the Wnt family (19 of which exist in humans), activating on the cell surface receptors of the Frizzled family (FZD, 10 found in humans) and LRP5/6 coreceptors [21]. After ligand-receptor interaction, FZDs transmit the signal in a GPCR (G protein-coupled receptor) manner [22,23,24,25], leading to dissociation of the β-catenin destruction complex. Cytoplasmic Dishevelled (DVL1-3) proteins act as intermediates between FZDs and the destruction complex [26]. Consequently, β-catenin accumulates in the cytoplasm and relocates to the nucleus where it acts as a transcription cofactor for TCF/LEF-dependent promotors, mostly activating the transcription of genes responsible for cell proliferation [21].

Wnt signaling can become aberrant in different manners in different types of disease. Insufficiency in the Wnt signaling compromises tissue renewal and underlies such deficits as osteoporosis, vitiligo, or neurodegeneration. Reciprocally, when overly active, it is the triggering cause of various types of cancer, most notably in the colon, stomach, liver, breast and ovaries [27,28]. Two principal routes to the overactivation of Wnt signaling can be applied in different cancers—mutational and epigenetic. The first one is dominant in e.g., colorectal cancers and hepatocellular carcinoma, with loss-of-function mutations in APC prevalent in the former, and gain-of-function mutations in β-catenin or loss-of-function mutations in Axin prevailing in the latter [29,30]. In contrast, breast cancer, especially its most aggressive triple-negative subtype, overactivates the Wnt pathway through dramatic disbalancing in expression levels of the pathway components, both positive and negative [31]. The hunt for Wnt signaling modulators as potential drugs has been open for many years, yet no therapeutics affecting the pathway have so far been approved, and only a handful have entered the early-phase clinical trials (in which some candidates miserably fail) [32,33,34]. These difficulties in finding therapeutics targeting the Wnt pathway are in sharp contrast to the advances made with other signaling pathways. These difficulties also call for expansion of the drug discovery approaches (so far mainly restricted to synthetic libraries and design of biologics) dedicated to the search of Wnt pathway modulators. NP are the ‘natural’ resort to look at for such an expansion. In this review, we summarize the existing data on activators and inhibitors of the Wnt pathway of natural origin, focusing on their source organisms, mechanisms of action, and drug development and therapeutic potential.

## 2. Wnt Activators from Natural Sources

Most of the artificial Wnt cascade activators known up to date, from “classic” inorganic lithium salts [35] to dozens of molecules obtained through chemical library screenings [36,37], in silico experiments [38,39], or peptide synthesis [40], have one particular enzyme as a target—the glycogen synthase kinase 3β (GSK3β). This kinase plays a crucial role in the function of the Axin-based β-catenin destruction complex. Inhibitors of GSK3β are sought for as drug candidates for various defects in skin, such as vitiligo [41] and androgenic alopecia [27,42], and in the bone, such as bone fracture [27] and osteoporosis [43]; other applications are also concievable. It is clear that novel molecules with GSK3β as the target are still in demand. What is the stand of NP research in this regard?

NP called indirubins discovered from edible mollusks [44] showed efficiency in inhibiting a series of kinases including GSK3β [45]. From that, an improved and more specific GSK3β inhibitor 6-bromo-indirubin-3′-oxime (6BIO) was synthetically derived, demonstrating neuroprotective activities during senescence [46]. Being initially discovered as metabolites of a gastropod but widespread in the living world (throughout invertebrates, plants, yeasts and bacteria) [47,48], indirubins and their derivatives, like 6BIO, are the “gold standards” in experiments related to Wnt signaling activation [49], alongside lithium chloride. The medchem optimization of indirubins continues, yielding more products with improved characteristics, such as indirubin-5-nitro-3′-oxime (INO) [50], as does the search for other GSK3β inhibitors from natural sources. Andrographolide, a labdane diterpenoid produced by the plant *Andrographis paniculata*, is a potent Wnt signaling activator acting by inhibiting GSK3β by a non-ATP-competitive, substrate-competitive mode of action. In silico analyses suggested that the compound interacts with the substrate-binding site of GSK3β [51]. A screen of 15 Chinese traditional medicinal herb extracts, based upon an enzyme-immobilized magnetic beads binding assay combined with high-performance liquid chromatography, identified three potential GSK3β inhibitory activities in the plants *Euonymus fortunei, Amygdalus communis*, and *Garcinia xanthochymus*. The active substance from the latter plant was isolated as the flavone fukugetin, inhibiting GSK3β with IC_50_ = 3.2 μM. Enzyme kinetics and molecular docking experiments further unveiled the mechanism of action, revealing fukugetin as a non-ATP competitive inhibitor interacting with the phosphate recognizing substrate binding site of GSK3β [52].

The inhibition of GSK3β is a powerful and robust but often unprecise instrument—some of the GSK3β inhibitors listed above can also block the action of other kinases such as GSK3α [45]. Despite enthusiasm surrounding the GSK3β inhibitors as Wnt pathway activators, one has to be aware that GSK3β is implicated in many other critical cellular functions including cell cycle control, proliferation, differentiation and apoptosis [53] and any Wnt-activating drug targeting this enzyme has a high potential to cause adverse effects. The same can be stated about the second kinase of the β-catenin destruction complex, CK1 [54,55], which does not scare away the interest in developing compounds targeting this enzyme [55]. As an NP example, ricinine, a pyridone alkaloid from *Ricinus communis*, showed the ability to stimulate the Wnt cascade, having the CK1 as the target [56].

Recent studies tend to strive towards screening of libraries of naturally derived compounds and extracts with up-to-date Wnt-relevant cell-based assays. Such a screening of 83 Korean plant extracts revealed an ability of the extract of the *Sanguisorba officinalis L.* grass to activate the Wnt pathway in the classical TOPFlash transcriptional assay, and subsequently to control adipo-osteogenic differentiation, making it potentially useful for medicinal purposes in osteoporosis as well as obesity treatment [57]. Another study performed TOPFlash-based screening of 350 aqueous plant extracts identifying the extract of the *Hovenia dulcis* tree to activate Wnt signaling, to induce osteogenic differentiation of murine calvarial osteoblasts without being cytotoxic, and to increase femoral bone mass without affecting mouse body weight [43]. This study went further, to identify the active component of the extract as methyl vanyllate. This compound could reproduce all the effects of the original extract and even showed a therapeutic effect comparable to that of parathyroid hormone (PTH)—the only anabolic agent approved by the FDA for osteoporosis treatment [58]—in a model of osteopenia in ovariectomized mice [43]. The nature-derived methyl vanyllate may have advantages over PTH: methyl vanyllate can be administered orally unlike intravenously injectable PTH, plus PTH has shown a carcinogenic potential [59]. Unfortunately, the published studies on methyl vanyllate do not focus on the molecular target(s) and the mechanism(s) of action.

As suppression of osteogenesis and induction of bone regeneration are controlled by the Wnt pathway activation, a large series of works link the capacity of a plant extract or its components to show positive effects in cell- or animal-based bone disease models with stimulation of Wnt signaling. For example, l-quebrachitol (2-*O*-methyl-l-chiro-inositol), a naturally occurring methoxy analog of inositol found in many plants and recoverable as a byproduct from the rubber industry, could promote osteogenesis in a pre-osteoblastic MC3T3-E1 cell model. Increased expression of the Wnt pathway components LRP5, β-catenin, Wnt5a, and FZD4 was observed during this experiment. These findings cumulatively brought the authors to conclude that l-quebrachitol acted as a potential Wnt pathway activator, the exact mechanism remaining unknown [60]. As another example, experiments with ovariectomized rats demonstrated that extracts of the Chinese medical herb *Rehmannia sp.* [61] promoted improvements in bone parameters in experimental animals. Concomitantly, downregulation of the Wnt inhibitors DKK1 and SOST was observed, along with the decreased ratio of phosphorylated (inactive) β-catenin to total β-catenin and the increased ratio of phosphorylated (inactive) GSK3β to total GSK3β in tibia and femurs. These findings suggested the activation of Wnt signaling, possibly achieved through decreasing the levels of Wnt inhibitor proteins [61]. Similar experiments by the same group and on the same model showed promotion of osteogenesis and Wnt pathway activation by extracts of *Salvia miltiorrhizae* [62]. Salvianolic acid B was isolated as the Wnt-activating component of *S. miltiorrhizae*, increasing amounts of nuclear β-catenin [63]. It is puzzling that extracts or compounds from the same plants were found by other researchers to downregulate the Wnt pathway: the glycoside fraction from *Rehmannia glutinosa* inhibited Wnt signaling [64], while dihydrotanshinone I isolated from *S. miltiorrhizae* also inhibited Wnt signaling, at the level of β-catenin, and suppressed osteosarcoma in cell line models [65].

A screen of 100 plant extracts identified extracts from *Euodia sutchuenensis Dode* leaves and young branches as active in stimulating osteoblast differentiation and enhancing murine calvarial bone formation ex vivo, via activation of the Wnt pathway as measured by increased levels and nuclear accumulation of β-catenin in murine primary osteoblasts [66]. Extracts of the widespread plant *Ginkgo biloba*, abundantly used in traditional medicinal practices, were able to stimulate osteogenic differentiation of human bone marrow mesenchymal stem cells through Wnt signaling. The β-catenin levels were elevated in the experimental cells upon treatment with the extract, and loss-of-function experiments proved that Wnt signaling was indeed involved in this model of osteogenesis [67]. Another study went further to identify at least one Wnt-stimulating component in *G. biloba*, namely a terpenic trilactone bilobalide. It activated Wnt signaling in P19 embryonic carcinoma cells leading to their neuronal differentiation, by the mechanism of GSK3β phosphorylation, but it also additionally increased the expression of Wnt1 and Wnt7a proteins, thus acting at different levels in a synergistic manner [68]. One more Wnt activator from the same plant turned out to be an alkaloid ginkgolide B, which upregulated the pathway in cell line and mouse osteoporosis models [69].

Another Wnt cascade activator was identified as kirenol, a natural diterpenoid compound isolated from plants of the *Siegesbeckia* genus using pre-osteoblastic MC3T3-E1 cells as a Wnt-dependent model of osteoblast differentiation. Kirenol treatment significantly upregulated mRNA levels of components of the Wnt pathway, including LRP5, DVL2 and β-catenin. In addition, kirenol efficiently upregulated β-catenin, inactivating GSK3β by stimulating its phosphorylation [70]. Guava triterpene-enriched extracts also revealed an osteoanabolic effect in ovariectomized rats, along with the activation of Wnt signaling by means of GSK3β phosphorylation [71]. Water extracts of *Morinda citrifolia* leaves enhanced the osteogenic differentiation of human periodontal ligament cells, activating Wnt signaling through increasing GSK3β phosphorylation and nuclear translocation and transcriptional activity of β-catenin, acting through the PI3K/Akt-dependent mechanism [72].

Another widely used plant metabolite, rosmarinic acid, activated Wnt signaling in a LacZ-based assay in cultured calvarial osteoblastic cells and stabilized cytoplasmic β-catenin in bone marrow-derived stroma ST2 cells [73]. An abundant NP, alpha-lipoic acid, improved osteogenesis in ovariectomized rats through the Wnt pathway as demonstrated by RT-PCR downregulation of the Wnt inhibitor DKK1 and upregulation of LRP5 [74]. Another example in this series is curculigoside, a phenolic glycoside and a metabolite of the *Curculigo orchioides* plant, which induced osteogenic differentiation of human amniotic fluid-derived stem cells, with the activation of Wnt signaling seen as upregulation of β-catenin [75]. Polysaccharides from the Chinese traditional medicinal plant *Bauhinia championi (Benth.)* from the genus *Bauhinia* (*Leguminosae*) promoted viability of chondrocytes isolated from rats, concomitant with the Wnt cascade upregulation seen as overexpression of Wnt4, β-catenin, FZD2 and cyclin D and the downregulation of GSK3β, both at the mRNA and protein levels [76]. Interestingly, even lipopolysaccharides from our common bacterial symbiont *Escherichia coli* showed the potential to activate Wnt signaling in odontogenesis in the case of the osteogenic differentiation of human periodontal ligament stem cells, where Wnt pathway in turn stimulated the action of a transcriptional osteogenic factor TAZ [77], thus providing an example of how our microbiota may coevolve with our signaling pathways and interplay with them.

In general, this large corpus of data obtained mostly in animal and cellular bone and cartilage models can lead us to several conclusions. Plants are a rich source of potential Wnt cascade activators, with many active compounds already isolated and characterized. Unfortunately, these publications focus mainly on preclinical studies of a therapeutic potential of compounds and extracts, typically leaving the mechanisms of action relatively unexplored. Regarding bone regeneration, the exact action mechanisms and/or targets related to Wnt signaling are in most cases unknown. Further, little can be deduced about tissue-specificity of the Wnt cascade activation by these extracts and compounds, studied in the bone-directed experiments. Activation of Wnt signaling might not be a direct consequence of a given compound, but a secondary effect caused by the cross-talk of Wnt signaling and other osteogenic pathways, suggesting that compounds increasing β-catenin levels in the bone might fail to do so in skin, liver, muscle or other tissues.

Not only bone and cartilage but also skin and hair are promising targets for the Wnt-targeting regeneration therapy. Aiming at a new hair growth-promoting drug, a screen of 800 extracts was conducted using the TOPFlash assay in HEK293 cells, and hits were tested for the ability to promote hair growth in cell-based and mouse models. Extracts from *Aconitum ciliare* were among the most active both in the TOPFlash and hair growth assays, positioning this medicinal plant for the identification of a promising Wnt activator [78]. Flavonoids from the plant *Vernonia anthelmintica* and their derivatives emerged as Wnt pathway activators for melanin synthesis in vitiligo—a sickness manifested as skin depigmentation [41]. As a potential mechanism, phosphorylation and thus inhibition of GSK3β via crosstalk with the PI3K/Akt pathway was pinpointed [41]. In this context, it is interesting to mention that tobacco smoking-caused skin pigmentation was shown to increase β-catenin levels, the activity linked to the tobacco plant (*Nicotiana tabacum*) metabolites [79].

In the nervous tissue, activation of the Wnt cascade was demonstrated by cannabidiol, a non-psychotomimetic phytocannabinoid from the *Cannabis sativa* plant. Specifically, cannabidiol upregulated Wnt signaling in human neural PC12 cells by deactivating GSK3β, thus delivering a neuroprotective activity in a cellular model of Alzheimer’s disease [80]. In another example, oral administration of biomass of a medicinal mushroom *Coriolus versicolor* increased dendritic length and dendritic branching, concomitant with increased cytonuclear β-catenin in mouse hippocamp [81].

Genistein, an isoflavonoid isolated from soybeans, was described to interact with plasma membrane and nuclear estrogen receptors [82], the former causing the ERK-mediated phosphorylation of GSK3β, thus leading to nuclear β-catenin accumulation, and the latter—stimulating co-activators of β-catenin (P300/CBP, PACF, SMARCD1) to facilitate expression of Wnt target genes in the adipose tissue [83]. These findings led the authors to propose isoflavones as perspective regenerative agents against non-alcoholic fatty liver disease [83]. Flavonoids from the Chinese traditional medicinal plant *Herba epigenii* enhanced osteogenic differentiation of human bone marrow-derived mesenchymal stem cells through the upregulation of Wnt signaling measured as increased β-catenin and enhanced expression of a set of Wnt target genes [84]. A flavonoid calycosin, also a plant-derived product related to traditional Chinese medicine, activated the Wnt pathway in HCT116 and LoVo human colorectal cancer cells, as seen by nuclear translocation of β-catenin [85]. Berberine, a benzylisoquinoline plant alkaloid from *Coptidis rhizoma*, promoted osteogenic differentiation of bone mesenchymal stem cells, concomitant with accumulation of total and nuclear β-catenin; activation of the Wnt cascade was also confirmed by the TOPFlash assay. Furthermore, the Wnt signaling inhibitor DKK1 was blocked by berberine [86]. A polysaccharide fraction from the plant *Achyranthes bidentata* used in the traditional Chinese medicine for the treatment of ostheoarthritis was shown to promote proliferation of rat chondrocytes by activation of the Wnt pathway, seen as upregulation of Wnt4, FZD2, β-catenin and cyclin D, and downregulation of GSK3β expression; nuclear translocation of β-catenin was also observed [87]. Similarly, 2,4,5-trimethoxyldalbergiquinol isolated from the medicinal plant *Dalbergia odorifera* promoted differentiation of mouse osteoblasts in culture, involving the activation of Wnt signaling detected at the level of increased mRNA expression of Wnts, phosphorylation of GSK3β, and nuclear accumulation of β-catenin [88]. In all these cases, an extract or an active compound thereof is found to trigger osteogenic or chondrogenic processes through activation of the Wnt cascade; however, the exact molecular target remains unclear.

A pioneering work in NP research and biology-oriented chemical synthesis gave rise to a new class of small molecule co-activators of the Wnt cascade [89]. The authors used NP with various known bioactivities like a marine-derived antitumor agent sodwanone S [90], plant-derived allelopathic and phytotoxic hellianuols [91], and a contraceptive zoapathanol [92] to design multifarious oxepane scaffolds resembling the core scaffolds of bioactive natural products, thus creating diversity while mimicking the nature. This approach led to generation of NP-related libraries, which could be screened for bioactivities including the activation of the Wnt cascade. The cell-based TOPFlash assay identified a series of structurally related oxepanes, which could serve as co-activators of the Wnt cascade in the presence of the canonical Wnt3a ligand. Subsequent pull-down and mass-spectrometry experiments revealed the mechanism of the co-activation: oxepanes bound to the van-Gogh-like receptor protein 1 (Vangl1) [93]. Vangl1 had earlier been reported as a negative modulator of the canonical Wnt signaling [94,95]. The putative mechanism of the oxepane action was that the compounds liberated and restored the signal transducing activity of the crucial Wnt pathway component DVL, which was impaired by Vangl1 [89]. In general, this elegant study provides an example of sophisticated manipulations with NP creating new analogs with novel properties and yielding Wnt pathway activators different from the GSK3β inhibitors.

The classical GSK3β inhibitors indirubins were derived from mollusks. To continue the exploration of marine NP targeting Wnt signaling, recently a TOPFlash-based screening of 81 ethanol extracts from deep-sea invertebrates dwelling in the Kuril Basin of the Pacific Ocean revealed a series of Wnt modulating activities. Of special interest among the examined species are a holothurian *Molpadia musculus*, a polychaeta *Travisia sp.*, and different representatives of the order *Actiniaria* including *Phelliactis callicyclus*, which were able to (co)activate the Wnt cascade [96]. Given the superficial nature of the screening, little can be said about the active components and their targets, but the mere fact that a single screen of deep-sea organisms gave a variety of potential Wnt activators belonging to different invertebrate orders calls for more attention to marine organisms as a source of biomolecules for future targeted therapies. The discovery of Wnt activating activities in marine organisms continues; one of most recent examples is activation of Wnt signaling by a fermented extract of a pacific oyster *Crassostrea gigas*. It suppressed ovariectomy-induced osteoporosis and osteoclastogenesis in mouse models, and activated osteogenesis in cell cultures and zebrafish larvae, acting through β-catenin-dependent transcription [97].

Algal metabolites, most notably polysaccharides, also displayed a Wnt-activating capacity. Extracts of *Undariopsis peterseniana*, edible brown algae, were tested in hair growth models in a search for drug candidates against androgenic alopecia and found to stimulate ex vivo hair-fiber growth in rat vibrissa follicles as well as in vivo hair growth in mice. It showed an increase in β-catenin accumulation and GSK3β phosphorylation in dermal papilla cells [98]. A polysaccharide fraction from green algae *Capsosiphon fulvescens* was able to promote growth of rat gastrointestinal IEC-6 cells, involving activation of MAPK and Wnt signaling, the latter confirmed by the analysis of the Wnt target genes cyclin D and c-myc and by nuclear translocation of β-catenin [99]. Similarly, hot water extracts from unicellular microalgae *Chlorella vulgaris* promoted proliferation of IEC-6 cells, with activation of MAPK, PI3K/Akt and Wnt pathways, the latter seen as increased nuclear β-catenin and cyclin D expression [100].

The following conclusions can be drawn about the current state of NP activators of the Wnt pathway. Marine-derived indirubins and their derivative 6BIO remain the gold standard activators with GSK3β as the target. Alongside, a large pool of data describes activation of the Wnt signaling by plant extracts or metabolites in osteogenesis models without knowledge of the exact molecular mechanisms. In the cases where certain mechanistic investigation was conducted, GSK3β again emerged as the potential target, given the described effects of the NP on the phosphorylation status of this kinase. As discussed above, targeting this enzyme may produce undesirable side effects, thus the search for other means to activate the Wnt pathway, e.g., through specific activators of FZD receptors, is desired. With the high chemical diversity of the discovered Wnt activators (indirubins, terpenoids, flavonoids, pyridones, esters, polyatomic alcohols, lactones, polyphenoles, glycosides, lypopolysaccharides—see Table 1), one might expect that novel specific Wnt activators will be identified, provided that appropriate screening methods focusing on the upper levels of the pathway are applied [8,34]. It is also conceivable that the sources of potential NP Wnt activators, relatively poorly exploited so far (marine organisms, fungi and bacteria), may provide the desired novel types of the activators.

## 3. Wnt Inhibitors from Natural Sources

Given the paramount importance of excessive activation of the Wnt pathway in etiology of various cancers, much effort is given to the search for inhibitors of the pathway; NP are a natural source of chemodiversity to look for such inhibitors [101,102]. Quercetin was among the first NP Wnt inhibitors identified: this flavonoid abundant in many plants showed the ability to inhibit Wnt-dependent transcription, acting at TCF-based transcription complexes—the very downstream level of the cascade [103]. Quercetin was further shown to inhibit proliferation of Wnt-dependent cancer lines: SW480 cells of colon cancer [104], NT2/D1 cells of teratocarcinoma [105], and to reduce viability and epithelial-mesenchymal transition in prostate cancer PC-3 cells [106]. In also reduced survival and proliferation of B-lymphocytes (B1 cells) in vitro, which require Wnt signaling for proper development [107]. However, there are also some controversial findings that quercetin reduced lipopolysaccharide-induced apoptosis of osteoblasts and re-activated the Wnt pathway in these cells through elevation of expression levels of Wnts and β-catenin; it was proposed that such re-activation could be a compensatory mechanism in osteoblasts in response to the initial suppression of the Wnt pathway by quercetin [108]. To add to the controversy, quercetin was found to weakly activate TCF/LEF-mediated transcription without stimulation of cell growth [109]. One may propose that the interaction of quercetin with TCF/LEF and associated transcription factors may not always be inhibitory but sometimes stimulatory for the transcriptional activity. Quercetin, as a Wnt pathway inhibitor, was found to be efficient in co-treatment with the chemotherapeutic drug doxorubicin in mouse models of gastric carcinoma [110]. Wnt-inhibiting activities were also shown by a related flavonoid isoquercetin in colon cancer cells (SW480, DLD-1, and HCT116), again suggesting that the molecular target of the NP lies in the nuclear part of the Wnt signaling cascade [111].

Curcumin is another famous NP Wnt signaling inhibitor of plant origin. It is a diarylheptanoid isolated from turmeric (*Curcuma longa*), sold as a herbal supplement, cosmetics ingredient, food flavoring, and food coloring ingredient [112]. It was initially shown to arrest growth of HCT-116 colon cancer cells leading to β-catenin degradation, with no further clarifications of the mechanism of action [113]. Since then, numerous reports appeared on curcumin as a Wnt pathway inhibitor in various cell and animal models. Downregulation of the Wnt pathway by curcumin was observed in MCF-7 and MDA-MB-231 breast cancer cells [114]. It was also shown in osteosarcoma cells, through reducing the amount of nuclear β-catenin and leaving the level of cytoplasmic β-catenin unaffected [115]; another NP PKF118-310 was also described to act in the same manner in this work. Curcumin suppressed proliferation of prostate cancer cells decreasing the levels of TCF4, CBP and p300—transcriptional cofactors of β-catenin [116]. Similar results were obtained in colon cancer cells treated with natural analogs of curcumin: demethoxycurcumin, bisdemethoxycurcumin, and a metabolite of the latter, tetrahydrocurcumin, demonstrating that these compounds downregulated the β-catenin nuclear cofactor p300 [117]. These data cumulatively suggest that curcumin and its analogs, similarly to quercetin, are nuclear level inhibitors of the Wnt pathway. Curcumin was also found to decrease the rate of migration and proliferation of Hep3B hepatocarcinoma cells through inhibition of the Wnt signaling pathway [118,119], similarly to its effect in a medulloblastoma cell line [120], in glioblastoma [121], and in a non-small-cell lung cancer cell line A549 [122]. It suppressed corneal neovascularization through Wnt inhibition [123]. It suppressed the Wnt along with the Sonic Hedgehog pathway in lung cancer stem cells [124]. It suppressed Wnt signaling in gastrointestinal cancer cell lines [125,126]. This large corpus of data presents curcumin as a robust downstream-acting inhibitor of Wnt signaling with low tissue specificity.

However, curcumin was also reported to activate the Wnt pathway in some instances. It could suppress adipogenesis in a model of differentiation of 3T3-L1 cells into adipocytes, stimulating Wnt signaling by means of enhancing nuclear translocation of β-catenin. In parallel, curcumin reduced the differentiation-induced expression of critical components of the β-catenin destruction complex CK1, GSK3β, and Axin. Quantitative PCR analysis further revealed that curcumin increased the expression of Wnt10b, FZD2, and LRP5. Thus, it was concluded that curcumin stimulated Wnt signaling during adipogenesis [127]. In another study, curcumin also acted as a Wnt activator, downregulating GSK3β and increasing the β-catenin in neuroblast SH SY5Y cells [128]. Similarly, curcumin restored the expression of Wnt pathway components, leading to the nuclear translocation of β-catenin, and the rescuing of dexamethasone-induced osteoporosis [129]. These examples indicate that in the cases of Wnt signaling stimulation, curcumin acts through modulating the expression of key pathway elements, rather than affecting the nuclear activity of the pathway. The clue to this riddle can lie in the fact that curcumin may interact with various microRNAs and thus interfere with gene expression. Indeed, curcumin was reported to attenuate the levels of miR-17-5p, which in turn downregulated the expression of TCF7l2—a Wnt pathway effector. Normally, miR-17-5p upregulates adipogenic differentiation, and curcumin was able to suppress this process via activating the Wnt pathway through the microRNA-mediated TCF7l2 upregulation in 3T3-L1 pre-adipocytes, highlighting curcumin as a potential anti-obesity agent [130]. In another example, curcumin inhibited proliferation of oral squamous cell carcinoma SCC-9 cells through upregulation of miR-9, leading to increased GSK3β levels and thus suppression of Wnt signaling [131]. In a colon cancer mouse xenograft study using SW480 cells, curcumin reduced the tumor growth by suppressing the Wnt pathway through downregulation miR-130a [132].

As the clinical use of curcumin is limited due to its low potency and poor pharmacokinetic profile, attempts to optimize this compound have been performed, resulting in a series of derivatives with 6 to 60 times more potent Wnt inhibitory potential [133]; however, none of these analogs have yet been brought to clinical trials. In general, curcumin can be regarded as a low-selective downstream Wnt inhibitor active in most tissues. However, it additionally can modulate (in the suppressing or activating manner) the pathway through microRNAs, adding further complexity to the potential of this NP as a drug candidate.

Another abundant and broadly used inhibitor of Wnt signaling is a widespread phytoalexin of a polyphenolic nature—resveratrol. Similarly to curcumin, it is a downstream inhibitor on the pathway: resveratrol downregulates TCF4 crucial for the Wnt-induced transcription, probably through the stimulation of proteasomal degradation this transcription factor [134]. Resveratrol demonstrated the Wnt-inhibiting potential in many models and tissues: in HT29 and RKO colon cancer cells [135]; in glioma cells, impairing their proliferation and motility [136] and restoring their sensitivity to temoxolamide [137]; in MGC-803 gastric cancer cells, inhibiting their growth, as well as in Colo16 squamous cell carcinoma cells [138]; in U2-OS osteosarcoma cells, concomitant with increased connexin43 expression, known to additionally downregulate Wnt signaling [139]; in human induced pluripotent stem cells, enhancing their differentiation towards cardiomyocytes [140]. Resveratrol also showed its Wnt-inhibiting activity in an animal model (collagen-induced arthritis of Wistar rats), leading to the amelioration of inflammatory arthritis [141].

Resveratrol was also shown to fine-tune various signaling pathways including Wnt by regulating microRNAs, thus again resembling curcumin [142]. Further, resveratrol can affect long non-coding RNAs, such as NEAT1m whose downregulation by the polyphenol suppressed Wnt signaling and thus inhibited proliferation, migration and invasion of multiple myeloma cells [143]. Clinical trials failed to show the potency of resveratrol to block Wnt signaling in colon cancer, while the Wnt pathway was suppressed by the treatment in healthy colonic mucosa [144]. In general, quercetin, curcumin and resveratrol can be characterized as a “classic” trio of robust downstream Wnt pathway inhibitors, showing Wnt-downregulatory activity in a wide array of tissues. Beside their “nuclear” inhibiting function, they are sometimes reported to modulate the Wnt signaling in more sophisticated way by up- or downregulating non-coding RNAs affecting the pathway. In spite of large body of investigations and some clinical trials [144,145], these NP or their derivatives have not yet made it to approval as drugs.

Plant-derived substances are continuously studied for their ability to target components of the Wnt pathway. Some of them, similarly to quercetin or curcumin, affect the pathway at the nuclear transcription level: Emodin, an anthraquinone present in roots and bark of several medicinal plants, inhibits Wnt signaling in human colorectal cancer cells (SW480 and SW620) by downregulating the transcriptional co-activator p300 and upregulating the transcriptional repressor HBP1 [146]. Periplocin extracted from bark of the medicinal plant *Periploca sepium* induced apoptosis of SW480 cells, inhibiting Wnt signaling by reducing the affinity of the transcriptional TCF complex to its specific DNA binding sites [147]. Henryin, an ent-kaurane diterpenoid isolated from the medicinal plant *Isodonrubescens var. lushanensis* and used to prevent gastrointestinal diseases, inhibited proliferation of human colorectal cancer cells HCT116, downregulating the Wnt cascade by impairing association of the β-catenin/TCF4 transcriptional complex, likely through directly blocking the binding of β-catenin to TCF4, while cytoplasmic and nuclear levels of β-catenin are not affected [148]. Mangiferin, a natural compound of *Mangifera indica L.*, was shown to inactivate the Wnt pathway in human hepatocellular carcinoma cells MHCC97L and HLF downstream of β-catenin, downregulating the LEF1 coactivator protein WT1 [149]. Ginkgetin, a biflavone isolated from the plant *Cephalotaxus fortunei*, inhibited the Wnt pathway in medulloblastoma cells, not affecting β-catenin amounts, possibly hinting at a nuclear target of this compound [150].

Other modes of action of plant-derived Wnt pathway inhibitors have also been described. Cardamonin, a chalcone, showed antitumor activity in breast cancer cells (MCF-7, BT-549, MDA-MB-231 and others) and in murine breast tumor models, leading to tumor growth arrest, apoptosis and inversion of the epithelial-mesenchymal transition. The luciferase-based TOPFlash assay identified cardamonin as a Wnt pathway inhibitor acting at GSK3β: through inhibition of the deactivating phosphorylation of GSK3β by Akt, cardamonin restored the activity of the former leading to increased β-catenin destruction [151]. A screening of a plant extract library combined with bioinformatical methods of metabolite bioactivity prediction followed by isolation of prioritized metabolites was performed, yielding several Wnt-inhibiting compounds, such as deoxyphorbol esters from extracts of the shrub *Bocquillonia nervosa* and the tree *Neoguillauminia cleopatra* [152]. Some NP affect Wnt signaling at the level of non-coding RNA or mRNA translation, such as (−)−gossypol, a polyphenol from cottonseed [153], and its derivative Gossypolone [154], which were characterized as inhibitors of Musashi-1 (MSI1), an RNA-binding protein that acts as a translation activator of target mRNAs. One of MSI1 targets is the mRNA for APC that downregulates Wnt signaling. Inhibiting MSI1 by (−)−gossypol led to Wnt downregulation and suppressed human colon cancer cell lines HCT-116, HT-29, DLD-1 and LS174T [153]. Another potential tumor growth inhibitor downregulating the Wnt and several other signaling pathways is a plant-derived flavonoid apigenin, which acted through regulatory microRNAs [155]. Loureirin B, component of the Chinese traditional herb *Sanguis Draxonis*, inhibited proliferation and promoted apoptosis of rat hepatic stellate cells by downregulating Wnt signaling, seen as decreased expression of Wnt1 and β-catenin. The compound also upregulated miR-148-3p, and knockdown of this microRNA reversed the effects of loureirin B [156].

Some other NP inhibit Wnt signaling by as yet unknown mechanisms. For example, the plant-derived polycyclic compound dihydroartemisinin used as an anti-malaria drug, was shown in a drug repositioning trial to suppress growth of squamous cell carcinoma A431 cells by downregulating the Wnt pathway [157] acting at the level of upregulation of phosphorylated β-catenin [158]. Extracts of Jerusalem artichoke (*Helianthus tuberosus*) and the Far Eastern endemic liana *Ampelopsis japonica* inhibited Wnt signaling in cell-based GFP assays and in the TOPFlash assay [159], the exact mode of action and the active components remaining to be identified. An extract of *Telectadium dongnaiense*’s bark and its active component periplocin inhibited Wnt signaling in the TOPFlash assay in human colon carcinoma HCT116 cells and reduced expression of a series of Wnt target genes [160]. A plant-derived isoquinoline alkaloid berberine and its synthetic 13-arylalkyl derivatives reduced the levels of cytoplasmic β-catenin in HCT116 human colon carcinoma cells [161]. Ethanol extracts of the *Scutellaria barbata* plant reduced colorectal cancer cell (HT-29) xenograft growth in mice, and downregulation of Wnt signaling in the tumors was verified at the protein level of expression of Wnt target genes c-myc, survivin and APC, and β-catenin itself as well as β-catenin phosphorylation levels [162]. Extracts from two common south Italy apples, *Malus pumila Miller* cv. ‘Annurca’ and *Malus domestica* cv ‘Limoncella’, decreased Wnt signaling in cells carrying APC mutations upregulating the cascade and in ex vivo biopsies of patients with colorectal cancer cells carrying APC mutations. The effect was observed using the TCF-GFP constructs and β-catenin immunostaining. The authors suggested polyphenols, particularly quercetin and its glycosides, as the active substances of the extracts. Unfortunately, apple extracts as food supplements administered to patients did not produce any effect, probably due to loss of polyphenols in the digestive tract, leading to the recommendation by the authors to use enteric coating for future applications of these extracts as food supplements [163]. β-elemene, an active component of the traditional Chinese medicinal herb *Curcuma zedoaria*, was shown to slow down proliferation and migration of human cervical cancer SiHa cells, decreasing levels of β-catenin and TCF7 [164]. Gigantol, a bibenzyl compound derived from several medicinal orchids, suppressed viability and migratory capacity of MDA-MB-231 and MDA-MB-468 breast cancer cells. Western blotting showed that gigantol reduced the levels of phosphorylated LRP6, total LRP6 and cytoplasmic β-catenin in a dose-dependent manner, resulting in a decrease in expression of the Wnt target genes Axin2 and survivin [165]. γ-Tocotrienol, a compound naturally found in different vegetable oils, was shown to downregulate Wnt signaling in human colon carcinoma HT-29 cells [166]. Later, it was shown that a succinate ether derivative of tocotrienol inhibited Wnt signaling in malignant mesothelioma cells through epigenetic induction of expression of the Wnt antagonist DKK1, shedding light on the mechanism of action of the compound [167]. Honokiol, a bioactive constituent from the Magnolia plant, suppressed migration of human non-small cell lung cancer cells in a Wnt-dependent manner. Honokiol inhibited the Wnt pathway by increasing the levels of CK1α and GSK3β, and decreasing the cytoplasmic and nuclear β-catenin levels [168]. Hydnocarpin, a natural lignan isolated from the plant *Lonicera japonica*, was shown to suppress Wnt signaling in human colon cancer cells SW480, remarkably increasing the cytoplasmic levels of Axin, the scaffolding protein of the β-catenin destruction complex [169]. A slight downregulation of the Wnt pathway (along with some other oncogenic pathways) was shown using the TCF-GFP reporter in human breast cancer cell lines T47D and MCF7 by a mixture of bergamot-derived natural products Brutieridin and Melitidin [170]. *Gynura divaricata subsp. formosana* suppressed human hepatocellular carcinoma Huh7 cell growth, as well as xenograft hepatocellular carcinoma tumor growth by downregulating Wnt signaling, as seen at a panel of Wnt target genes and β-catenin [171]. Treatment of human colon carcinoma HT-29 cells with a water extract of a mixture of domestic rice (*Oryza sativa*) broken seeds, rice bran, and rice germ (so called “brewer’s rice”) inhibited Wnt signaling through upregulation of CK1 and *APC* mRNA [172]. Based on measuring the β-catenin expression by qPCR and immunoblotting in a rat model of azoxymethane-induced colon cancer, another study suggested that the active Wnt-inhibiting compound in rice could be inositol hexaphosphate [173].

A natural plant polyphenol rottlerin inhibited Wnt signaling in human prostate cancer PC-3 and DU145 cells and breast cancer MDA-MB-231 and T-47D cells through an interesting mechanism of reduction of phosphorylation (essential for activity) and protein levels of the Wnt coreceptor LRP6, not affecting LRP6 transcription [174]. Two main compounds from roots of the plant *Saussurea lappa*, dehydrocostus lactone and costunolide, inhibited the Wnt pathway and suppressed proliferation and survival of SW480 human colon cancer cells by the arrest of β-catenin translocation to the nucleus, not affecting cytoplasmic levels of β-catenin [175]. Similarly, bisleuconothine A, a bisindole alkaloid with an eburnane-aspidosperma type skeleton, decreased Wnt target gene expression in HCT116 and SW480 colorectal cancer cells through promoting the phosphorylation of β-catenin and the subsequent inhibition of its nuclear translocation; the NP also inhibited cell proliferation in vitro and dramatically suppressed tumor growth in HCT116 mouse xenografts [176]. Natural and semisynthetic tigliane diterpenoids from the plant *Euphorbia dracunculoides* inhibited Wnt signaling in a luciferase assay in HEK293 cells, reducing the expression of Wnt target genes Axin2, c-myc and cyclin D; phosphorylation and degradation of β-catenin were similarly observed in HEK293W cells incubated with tigliane diterpenoids [177]. *Euphorbiaceae* plants are otherwise famous for phorbol esters, of which several, of the New Caledonian origin, have produced strong Wnt-inhibiting activities in triple-negative breast cancer cells [152,178]. Similarly, diterpene esters—phorbol 12-myristate 13-acetate (PMA) and PEP005—isolated from *Euphorbia croton tiglium* and *Euphorbia peplus*, respectively, inhibited Wnt signaling in colon cancer cells [179]. The target of these phorbol esters was protein kinase C α (PKCα), which in turn phosphorylated β-catenin and RORα [179]. Coronaridine, an iboga type alkaloid from the plant *Tabernaemontana divaricata*, inhibited Wnt signaling in SW480 human colon cancer cells by decreasing β-catenin mRNA [180]. Vicenin-2, largely available from the medicinal plant *Ocimum sanctum*, and its apigenin form, 6,8-di-C-glucoside, inhibited viability of the human colorectal cancer HT-29 cells in the MTT assay by downregulating the Wnt pathway, monitored as decreased levels of β-catenin and cyclin D [181].

A plant-derived natural product parthenolide, a sesquiterpene lactone, inhibited Wnt signaling in the TOPFlash assay in HEK293 cells; further investigations showed that parthenolide did it by blocking the synthesis of the transcriptional factors TCF4/LEF1, probably performing it by binding the ribosomal protein L10, as siRNA knockdown of the latter also decreased TCF4/LEF1 levels and downregulated Wnt signaling [182]. The stabilization of mRNAs of Wnt pathway modulators is another example of inhibiting the Wnt pathway by flavonoids: the flavonoid EGCG ((−)−epigallocatechin-3-gallate), a major metabolite of many plants including green tea, downregulated the Wnt pathway by stabilizing mRNA of the HBP1 transcriptional repressor, which had previously been characterized as a suppressor of Wnt signaling; the data was obtained in MDA-MB-231 breast cancer cells [183]. A related polyphenolic compound, gallic acid, inhibited the Wnt pathway as seen by increased GSK3β and p-β-catenin levels and decreased p-GSK3β in B16F10 melanocyte cells. Moreover, a GSK3β-specific inhibitor (SB216763) restored gallic acid-induced melanin reduction in these cells [184].

1-Benzyl-indole-3-carbinol, a synthetic analogue of the natural phytochemical indole-3-carbinol derived from crucifer plants, suppressed Wnt signaling in a stronger and a more specific way than its natural precursors, shown by the downregulation of β-catenin protein levels with a concomitant increase in the levels of the β-catenin destruction complex components such as GSK3β and Axin. The compound was also active as an inhibitor in the TOPFlash assay, and the inhibitory effect was rescued by expression of a constitutively active form of LRP6, indicating that 1-Benzyl-indole-3-carbinol disrupted Wnt signaling at or upstream of LRP6 [185].

2-methoxystypandrone from the traditional Chinese medicinal plant *Polygonum cuspidatum* inhibited Wnt signaling in human triple-negative breast cancer cell lines, as measured by β-catenin levels and the TOPFlash assay, targeting the β-catenin destruction complex [186]. An intriguing and unexpected way of Wnt cascade downregulation in triple negative breast cancer cells, colon cancer cells and colon cancer organoids was shown by tannins from the Cameroonian medicinal plant *Syzygium guineense*. The tannins, compounds of a polyphenolic nature, revealed an ability to directly and selectively destabilize Wnt proteins in cell culture and in the colon cancer organoids, thus acting at the very upstream levels of the pathway. This Wnt protein destabilizing way of cascade downregulation may be very attractive for medicinal applications in tumors which arise due to the overproduction of Wnt ligands, and efficacy against the colon cancer organoids makes the *Syzygium guineense* plant an interesting candidate for a cancer-preventive food supplement [187].

The description above makes it clear that the major source of NP-based Wnt inhibitors (as is also the case for the activators) has so far been various medicinal plants. However, natural Wnt inhibitors have also been identified from other taxa, such as fungi. For example, a Wnt inhibiting activity in human lung adenocarcinoma cells was demonstrated by mycotoxin patulin isolated from the fungus *Penicillium vulpinum* [188]. Similarly, isopenicin, a meroterpenoid from the endophytic fungus *Penicillium sp. sh18*, showed Wnt inhibition in a dual luciferase assay in human colon cancer SW620 and HCT116 cells [189]. Anisomycin, an antibiotic produced by the fungus *Streptomyces griseolus*, selectively suppressed proliferation of leukemia cell lines and patient samples through suppressing Wnt signaling, as verified at the level of β-catenin and expression of a series of Wnt target genes [190]. Gliotoxin from the marine fungus *Neosartorya pseudofischeri* inhibited the growth of several Wnt-dependent colorectal cancer cell lines, downregulated Wnt signaling in the TOPFlash assay, and promoted degradation of β-catenin [191]. The mushroom *Ganoderma lucidum* used in traditional Chinese medicine blocked Wnt signaling through inhibiting the phosphorylation of LRP6 in human (MDA-MB-231) and mouse (4T1) breast cancer cell lines, also suppressing Wnt3a-activated expression of Axin2, a Wnt target gene [192]. Destruxin B, a cyclodepsipeptide from the entomopathogenic fungus *Metarhizium anisopliae*, suppressed proliferation and induced cell cycle arrest in human colorectal cancer HT29, SW480 and HCT116 cells suppressing Wnt-signaling by the downregulation of β-catenin and TCF4 protein levels and β-catenin/TCF4 transcriptional activity measured in the TOPFlash assay, concomitantly with decreased expression of the target genes cyclin D, c-myc and survivin. It also acted in vivo, suppressing tumorigenesis in HT29 xenograft mice; the on-target effect was confirmed by decreased levels of β-catenin, cyclin D, and survivin in the tumors [193]. Destruxin B demonstrated similar effects in hepatocellular carcinoma Sk-Hep1 cells, thus expanding it to other Wnt-dependent cancers [194]. Cordycepin (3′-deoxyadenosine) isolated from *Cordyceps sinensis*, a fungus parasitizing on larvae of Lepidoptera, inhibited the growth of B16-BL6 mouse melanoma cells by stimulating adenosine A3 receptors, which caused downregulation of the Wnt pathway upstream of GSK3β [195]. Bafilomycin, a specific inhibitor of the vacuolar proton ATPase that blocks endosomal acidification isolated from the fungus *Streptomyces griseus*, inhibited exogenous Wnt-stimulated, as well as DVL-stimulated signaling acting upstream of GSK3β. In HEK293 cells, it was shown that the mechanism involved the intracellular domain of LRP6 and vacuolar protein sorting protein 35, endosomal V-type ATPase, and endosomal trafficking [196]. Compounds isolated from the parasitic tree fungus *Inonotus obliquus* used in folk anticancer medicine inhibited Wnt signaling in human colorectal cancer cell lines HCT116, HT-29, SW620 and in mouse colorectal cancer models, involving the downregulation of nuclear β-catenin [197]. Compounds from the same fungus showed suppression of breast cancer growth in diabetic conditions in a rat model, also acting through the downregulation of β-catenin [198]. Interestingly, in the former study the compound responsible for Wnt downregulation was ergosterol peroxide [197], while in the latter it was claimed to be a lanostane triterpenoid inotodiol [198]. Such data indicate a possibility for synergistic anticancer Wnt-targeting action of the *Inonotus obliquus* extracts used in traditional medicine.

Some Wnt-inhibiting NP are not fungal- but lichen-derived, like caperatic and physodic acids isolated from *Platismatia glauca* and *Hypogymnia physodes* respectively, inhibiting Wnt signaling in HCT116 and DLD-1 colorectal cancer cell lines seen by the downregulation of Wnt target genes without affecting the levels or localization of β-catenin. The authors did not reveal the exact mechanism of action but proposed that the compounds interacted with nuclear transcription cofactors [199].

There is also information on Wnt inhibitors derived from prokaryotes: bioactive secondary metabolites of the aromatic and heterocyclic nature were isolated from terrestrial actinomycetes species and found to inhibit TCF/β-catenin transcriptional activity with the IC_50_ values of 0.6–7.4 nM [200]. Marine actinomycetes also yielded Wnt inhibitors, such as chromomycins A2 and A3 active in the TOPFlash assay [201].

Some Wnt-inhibiting small molecules can be found in more unexpected sources. For example, marinobufagin and other bufadienolides, cardiotonic steroids secreted by toads, demonstrated an ability to downregulate Wnt signaling acting at two levels: via GSK3β dephosphorylation at concentrations from 500 nM to 5 µM, and by an additional mechanism downstream from β-catenin stabilization, as shown in the TOPFlash assay with constitutively active β-catenin and TCF4 mutants [202]. However, these compounds are unlikely to be developed further as Wnt inhibitors due to their pronounced and unspecific cytotoxicity. Another study reported the Wnt-inhibitory potential of the venom of *Buthus martensi* scorpions. An analgesic peptide rBMK AGAP isolated from the venom was tested on breast cancer cell lines MCF-7 and MDA-MB-231, demonstrating inhibition of the cancer cell stemness, epithelial-mesenchymal transition, migration, and invasion in a series of assays as well as tumor growth reduction in xenograft mice. Downregulation of the Wnt pathway (along with NF-kB and the metastatic oncogene pentraxin 3) was involved as determined by decrease of β-catenin and p-GSK3β [203].

A few reports exist on the Wnt-inhibiting activities from marine sources. Two studies by the same group identified smenospongidine, a marine sponge-derived sesquiterpenoid quinone [204], and aeroplysinin-1, a brominated tyrosine derivative [205], as downstream inhibitors of the Wnt pathway. Both acted by promoting proteasomal degradation of β-catenin (induced by Wnt3a or a GSK3β inhibitor) and both affected expression of a series of Wnt target genes. More sponge metabolites of the sesterpenoid and steroid nature showed Wnt-inhibiting activities in luciferase assays [206]; sponge-derived quinones decreased β-catenin levels in HEK293 cells [207], identifying marine sponges as an interesting new source of potential Wnt inhibitors. TOPFlash-based high throughput screening of Pacific invertebrate extracts identified Wnt-inhibiting activities from holothurians *Peniagone sp.* and *Molpadia musculus*, from crustaceans *Calocarides quinqueseriatus* and *Munidopsis antonii*, from polychaetae, and especially from brittle stars (*Ophiuroidae*) including *Ophiura irrorata* [96]. These data suggest that Wnt inhibitors can be found in diverse taxa of marine organisms, not just the sponges highlighted in the prior studies.

Finally, like with the Wnt pathway activators above, the NP-inspired synthesis of small molecules yielded new inhibitors of the pathway. Analogs of withanolides, naturally occurring steroids built on the ergostane skeleton found mostly in the *Solanaceae* family of plants, were synthetized yielding a library of steroids including potent inhibitors of Wnt signaling, which acted upstream of the destruction complex, stabilizing Axin in a tankyrase-independent manner [208]. Another interesting example is clofazimine, a drug used to treat leprosy and some forms of tuberculosis, chemically synthesized based on the scaffold of riminophenazines isolated from lichens [209]. In a set of repositioning experiments, clofazimine emerged as a powerful inhibitor of Wnt signaling and proliferation in a panel of triple negative breast cancer lines in vitro and cell line-based and patient-derived mouse xenografts [210,211]. The drug acted at a yet unidentified (and potentially novel) nuclear or cytoplasmic component of the Wnt pathway. In mouse xenografts, clofazimine suppressed Wnt-mediated multidrug resistance and was compatible with conventional anticancer chemotherapies [211], serving as the basis for clinical repositioning trials of clofazimine against Wnt-dependent cancers currently in preparation. Suppression of Wnt-mediated multidrug resistance was also shown in a panel of cell lines with a series of flavonoids (theaflavin, quercetin, rutin, epicatechin 3 gallate and tamarixetin) previously known to downregulate Wnt signaling [212].

## 4. Discussion and Perspectives

Chemical diversity of the Wnt inhibitors is as broad as that of the activators (Table 1). The variety of mechanisms affected by NP-based inhibitors of the Wnt signaling pathway is higher than that of the pathway activators, but still, we can find some generalities. A large group of inhibitors acts on the very downstream levels of β-catenin interactions with its nuclear cofactors, like the best studied triade: quercetin, curcumin and resveratrol, and a series of their less-studied analogs. Another interesting phenomenon is that many natural compounds downregulate the Wnt pathway by affecting the expression levels of its key components by interacting with non-coding RNAs or even with ribosomal proteins [182]. Interestingly, the involvement of non-coding RNAs was much more often detected in case of natural inhibitors than activators. Another interesting target is at the other extreme of the pathway: the Wnt coreceptor LRP5/6, which is directly or indirectly targeted by many inhibitors [165,174,192,196]. In some cases, the inhibitors act through the induction of natural Wnt antagonists like DKK1 [167]. In many studies, the molecular mechanism is understudied or only roughly assessed, leaving the room for subsequent research. Intriguingly, several cases describe synergistic action against multiple actors within the Wnt pathway of multiple compounds present in the same extract [197,198], or even of a single compound having more than one molecular target [83]. Speaking of the NP with the identified mechanism of action, most target downstream levels of the pathway, thus being robust but unselective among the various Wnt signaling subtypes—the situation similar to that with natural Wnt activators mostly targeting GSK3β. However, the therapeutic development demands that small molecules capable to act on e.g., select, cancer-relevant FZDs or Wnt-FZD pairs are identified, as less side effects are expected with such drugs [9,34]. It is thus desired that future screens of Wnt pathway-targeting NP be conducted on more disease-relevant assay systems capable of distinguishing among different Wnt sub-pathways [8,9,34]. High-throughput screening platforms alternative to the standard TopFlash readout may also be desired [213].

**Figure 1:**
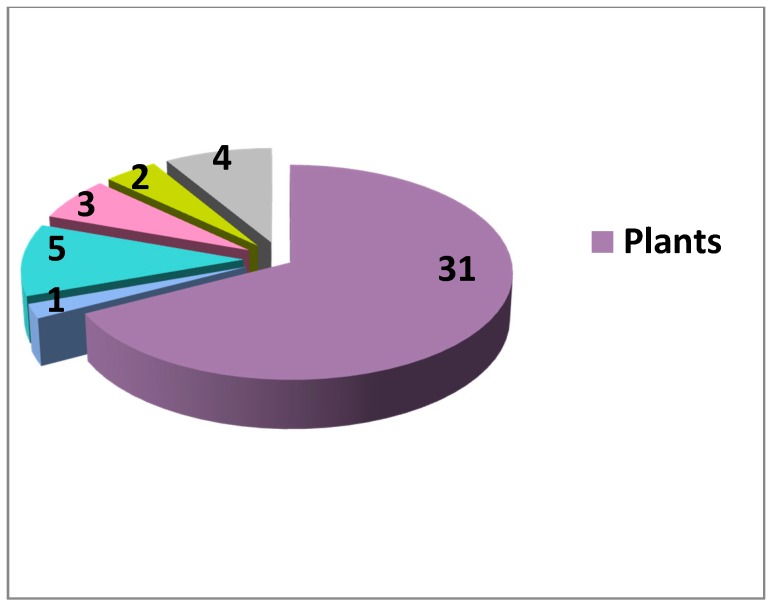
Distribution of known Wnt signaling activators from natural sources among different taxa.

**Figure 2:**
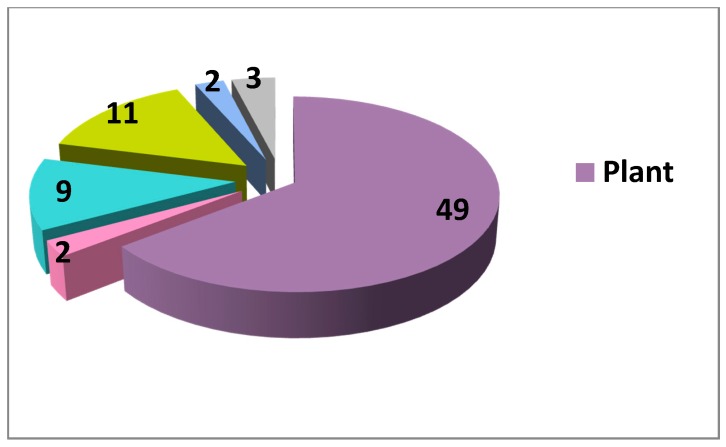
Distribution of known Wnt signaling inhibitors from natural sources among different taxa.

Concerning the sources of natural inhibitors and activators of the Wnt cascade, currently the vast majority originates from plants (Figure 1, Figure 2, Table 1). Although higher diversity exists for the inhibitors (with some fungal, prokaryotic and even vertebrate sources), there still exists an enormous under-representation of non-plant NP among Wnt modulators, asking for more research in these directions. Fungi are broadly used as anticancer agents in traditional medicine in many countries [214], thus being an attractive source for potential Wnt-inhibiting anticancer drugs. A rather simple screen of randomly picked marine invertebrates showed promising Wnt modulating activities in different taxa [96], highlighting the marine fauna and flora as another rich and almost unexplored source of small molecule regulators of the Wnt pathway and other therapeutically important cellular processes [13]. Protists, prokaryotes and other groups of organisms have also to be considered to yield new data on Wnt activators and inhibitors from natural sources and to pave the way to creation of novel drug candidates for the targeted treatment of Wnt-related cancers and for regenerative medicine. Many compounds affecting the Wnt pathway have been isolated and their mode of action identified (Table 1). On the other hand, a large pool of data exists on Wnt-modulating unseparated plant extracts or plant-derived compounds with unknown modes of action. More precise studies on these extracts and compounds is another clear direction for future research towards new drug candidates.

These considerations let us conclude this overview of the NP-based Wnt pathway activators and inhibitors with the following recommendations. First, the relatively poorly studied sources of NP as Wnt modulators—fungi, bacteria, and especially marine organisms—should be a major focus of future research. Second, aggressive pipelines of deconvolution of complex mixtures towards identifying the active compounds should be implemented to follow the screenings of crude extracts towards Wnt modulating activities. Finally, third, careful design and redesign of the screening platforms should be in place, in order to fine-tune the search towards identification of NP selectively acting at particular, disease- or organ-specific variants of the Wnt pathway, rather than bluntly activating or suppressing the Wnt signaling as a whole. These efforts should be accompanied with the prioritizing of R&D activities towards the NP with the highest chances to be developed as drug candidates, taking into account such parameters as in vivo potency, bioavailability, stability, and others relevant for the drug discovery, early on in the research on NP-based Wnt pathway modulators. We hope that these considerations will help the renewed interest in NP to deliver promising drug candidates targeting the Wnt signaling pathway in many diseases, from degeneration to cancer.

**Table 1:** Activators and inhibitors of the Wnt pathway from natural sources.

	**Activators**			
	Source organism	Acting substance	Mechanism/target	Reference
Plant	Plants (Indigo Naturalis, Isatis indigotica, Indigofera suffruticosa), mollusks (*Nucella lapillus*) and bacteria (*Providencia*, *Escherichia coli*, *Proteus mirabilis*, *Klebsiella pneumoniae*)	Indirubin	Inhibits GSK3β and GSK3α	[45,47,48]
		Synthetic derivative 6-bromo-indirubin-3′-oxime (6BIO)		[45,46,49]
		Synthetic derivative indirubin-5-nitro-3′-oxime (INO)		[45,50]
	*Andrographis paniculata*	Andrographolide (labdane diterpenoid)		[45,51]
	*Garcinia xanthochymus*	Fukugetin (flavone)		[45,52]
	*Ricinus communis*	Ricinine (pyridone alkaloid)	Inhibits GSK3β and CK1	[56]
	*Bauhinia championi*	Polysaccharides	Overexpression of Wnt4, β-catenin, FZD2 and cyclin D and downregulation of GSK3β at both mRNA and protein levels	[76]
	*Cannabis sativa*	Cannabidiol (phytocannabinoid)		[62]
	*Salvia miltiorrhizae*	Extract		[62]
	Siegesbeckia genus	Kirenol (diterpenoid)		[70]
	Guava fruit	Triterpene-enriched extract	Inhibits GSK3β	[71]
	*Ginkgo biloba*	bilobalide (terpenic trilactone)	-	[68]
		Ginkgolide B	-	[69]
		Extract	Increases β-catenin levels	[67]
	*Rosmarinus officinalis*, Lamiaceae, and Asteraceae family	Rosmarinic acid		[73]
	*Curculigo orchioides*	Curculigoside (phenolic glycoside)		[75]
	*Euodia sutchuenensis*	Extract		[66]
	*Coptis chinensis*	Berberine (alkaloid)	Increases total and nuclear β-catenin level	[86]
	*Cannabis sativa*	Cannabidiol	Inhibits GSK3β and DKK1	[80]
	*Achyranthes bidentata*	Polysaccharide fraction	Increases nuclear β-catenin	[87]
	*Salvia miltiorrhizae*	Salvianolic acid B	Inhibits GSK3β, increases nuclear β-catenin level	[63]
	*Dalbergia odorifera*	2,4,5-trimethoxyldalbergiquinol		[88]
	*Morinda citrifolia*	Extract	Inhibits GSK3β, increases β-catenin through PI3K/Akt	[72]
	*Vernonia anthelmintica*	Flavonoids	Inhibits GSK3β through PI3K/Akt	[41]
	*Aconitum ciliare*	Extract	β-catenin transcription	[78]
	Soybeans	Genistein (isoflavonoid)	Inhibits GSK3β via ERK (increases nuclear β-catenin)	[83]
	*Rehmannia sp.*	Extract	Downregulation of the Wnt inhibitors SOST and DKK, GSK3β phosphorylation	[61]
	*Sanguisorba officinalis*	Extract	-	[57]
	*Hovenia dulcis*	Methyl vanyllate	-	[43,58]
	Sapindaceae family, Elaeagnaceae family	l-quebrachitol (2-*O*-methyl-l-chiro-inositol) (methoxy analog of inositol)	-	[60]
	*Epimedium wushanense*	Flavonoids	-	[84]
	*Nicotiana tabacum*	Extract of cigarette tobacco	-	[79]
	Many plants	Calycosin	-	[85]
Algae	*Undariopsis peterseniana*	Extract	Increases of β-catenin accumulation and GSK3β phosphorylation	[98]
	*Capsosiphon fulvescens*	Polysaccharide fraction	Increases nuclear β-catenin level	[99]
	*Chlorella vulgaris*	Extract	-	[100]
Marine rtebratesorganisms	Holothurian *Molpadia musculus*	Extract	-	[96]
	Polychaete *Travisia sp.*	Extract	-	[96]
	Deep-sea anemone *Phelliactis callicyclus*	Extract	-	[96]
	Oyster *Crassostrea gigas*	Extract	-	[97]
Fungi	*Ganoderma lucidum*	β-glucan	-	[215]
	*Coriolus versicolor*	Extract	-	[81]
Bacteria	*Escherichia coli*	Lipopolysaccharides	-	[77]
Others	Plants, meat, milk, fungi	Alpha-lipoic acid	Downregulations of DKK1 and upregulation of LRP5	[74]
		Synthetic derivative oxepanes	Binds Vangl1 and restores the signaling activity of DVL	[89]
	**Inhibitors**			
	Natural sources	Compound	Mechanism/Target	Reference
Plants	*Syzygium guineense*	Tannins	Destabilize Wnt proteins	[187]
	different vegetable oils	γ-Tocotrienol (vitamin e)	Inductions of expression of DKK1	[166,167]
	*Mallotus philippensis*	Rottlerin (polyphenol)	Suppresses expression and phosphorylation of LRP6	[174]
	genus Magnolia	Honokiol (lignan)	Increases expression CK1α and GSK3β	[168]
	*Lonicera japonica*	Hydnocarpin (lignan)	Increases cytoplasmic levels of Axin	[169]
	Solanaceae family	Analogs of withanolides (steroids)	Stabilize Axin	[208]
	*Scutellaria barbata*	Extract	Reduces Wnt target genes and β-catenin	[162]
	*Berberis vulgaris*	Berberine (alkaloid) and its synthetic 13-arylalkyl derivatives	Reduces β-catenin	[161]
	Orchidaceae family	Gigantol (alkaloid)	Reduces phosphorylated LRP6, total LRP6 and cytoplasmic β-catenin	[165]
	*Saussurea lappa*	Dehydrocostus lactone	Arrests β-catenin translocation to nucleus	[175]
		Costunolide		
	*Leuconotis griffithii*	Bisleuconothine A (alkaloid)	Promotes phosphorylation of β-catenin and inhibits its nuclear translocation	[176]
	*Tabernaemontana divaricata*	Coronaridine (alkaloid)	Decreases β-catenin mRNA	[180]
	*Ocimum sanctum*	Vicenin-2 (flavonoid)	Decreases β-catenin	[181]
	*Cephalotaxus fortunei*	Ginkgetin (biflavone)	-	[150]
	Green and black tea, fruits, vegetables	Quercetin (flavonoid)	Suppresses binding within the TCF-based transcription factor complexes	[103,104,105,106,107]
		Isoquercetin (flavonoid)		[111]
		EGCG ((−)−epigallocatechin-3-gallate) (flavonoid)	-	[183]
		Gallic acid	Increases GSK3β and p-β-catenin	[184]
	Grapes, wine, cacao, hazelnuts	Resveratrol (stilbene)	Downregulates TCF4 by interacting with relevant microRNAs	[134,142,143]
	*Curcuma longa*	Curcumin (flavonoid)	Decreases the levels of TCF4, CBP and p300, reduces nuclear β-catenin	[115,116,117,130,131,132]
		Demethoxycurcumin	Downregulates p300 by interacting with relevant microRNAs	[117]
		Bisdemethoxycurcumin		
		Tetrahydrocurcumin		
	*Curcuma zedoaria*	β-elemene (sesquiterpene)	Decreases β-catenin and TCF7	[164]
	Cruciferae family	1-Benzyl-indole-3-carbinol (synthetic analogue of the natural phytochemical indole-3-carbinol)	Increases GSK3β and Axin, decreases β-catenin	[185]
	*Periploca sepium*	Periplocin (cardiac glycoside)	Reduces binding of TCF complex to specific DNA binding site	[147]
	*Telectadium dongnaiense*		-	[160]
	*Isodon rubescens var. lushanensis*	Henryin (diterpenoid)	Impairs association of β-catenin/TCF4 transcriptional complex	[148]
	*Tanacetum parthenium*	Parthenolide (sesquiterpene)	Synthesis TCF4/LEF1	[182]
	Gossypium genus	Gossypol (polyphenol)	Inhibitor of MSI1	[153]
		Gossypolone	-	[154]
	*Matricaria recutita*, *tanacetum parthenium*, citrus	Apigenin (flavonoid)	Regulatory microRNAs	[155]
	*Sanguis Draxonis*	Loureirin B (flavonoid)	Upregulates miR-148-3p	[156]
	*Artemisia annua*	Dihydroartemisinin	Increases phosphorylated β-catenin	[157] [158]
	*Malus pumila Miller cv. ‘Annurca’*	Extract	-	[163]
	*Malus domestica cv ‘Limoncella’*	Extract	-	
	*Mangifera indica*	Mangiferin	Downregulates the LEF1 coactivator protein WT1	[149]
	*Rehmannia glutinosa*	Glycoside fraction	-	[64]
	*Salvia miltiorrhizae*	Dihydrotanshinone	-	[65]
	*Helianthus tuberosus*	Extract	-	[159]
	*Ampelopsis japonica*	Extract	-	
	*Gynura divaricata*	Extract	-	[171]
	*Oryza sativa (rice)*	extract	Upregulates CK1	[172]
		Inositol hexaphosphate	-	[173]
	*Many plants*	Cardamonin	Inhibits the deactivating phosphorylation of GSK3β by Akt	[151]
	*Bocquillonia nervosa*	12-deoxyphorbol esters	-	[152]
	*Neoguillauminia cleopatra*			
	*Euphorbia dracunculoides*	Tigliane (diterpenoids)	-	[177]
	*Euphorbia croton tiglium*	PMA (diterpene ester)	PKCα (cross-talk with Wnt pathway)	[179]
	*Euphorbia peplus*	PEP005 (diterpene ester)	PKCα (cross-talk with Wnt pathway)	[179]
	*Citrus bergamia (bergamot)*	Brutieridin and Melitidin	-	[170]
	Plants: rhubarb, buckthorn and Japanese knotweed (*Reynoutria japonica*) Fungi: Aspergillus, Pyrenochaeta and Pestalotiopsis	Emodin (anthraquinone)	Interacts with TCF/LEF, downregulates p300, upregulates the repressor HBP	[146]
	*Polygonum cuspidatum*	2-methoxystypandrone	Targets β-catenin destruction complex, decreases β-catenin	[186]
	Solanaceae family	Synthetic derivatives of withanolides	Stabilize Axin	[208]
	Lichens (*Platismatia glauca*, *Cladonia uncialis*, *Parmelia sulcata*, *Hypogymnia physodes*, and *Hypocenomyce scalaris*)	Caperatic acid	-	[199]
Lichens	Lichens (*Platismatia glauca*, *Cladonia uncialis*, *Parmelia sulcata*, *Hypogymnia physodes*, and *Hypocenomyce scalaris*)	Physodic acid	-	[199,210,211]
		Synthetic riminophenazine derivative: Clofazimine	-	
	*Penicillium vulpinum*	Patulin (mycotoxin) polyketide	-	[188]
Fungi	*Penicillium sp. sh18*	Isopenicin (meroterpenoid)	-	[189]
	*Streptomyces griseolus*	Anisomycin (antibiotic)	Decreases β-catenin	[190]
	*Ganoderma lucidum*	Extract	Inhibits phosphorylation of LRP6	[192]
	*Metarhizium anisopliae*	Destruxin B (mycotoxin) cyclodepsipeptide	Downregulates β-catenin and TCF4 and β-catenin/TCF4 transcriptional activity	[193,194]
	*Cordyceps sinensis*	Cordycepin (3′-deoxyadenosine)	Stimulates adenosine A3 receptors	[195]
	*Streptomyces griseus*	Bafilomycin (antibiotic)	Acts upstream of GSK3β	[196]
	*Neosartorya pseudofischeri*	Gliotoxin	Degradations of β-catenin	[191]
	*Inonotus obliquus*	Ergosterol peroxide (Steroid)	Decreases nuclear β-catenin	[197]
	*Inonotus obliquus* *Actinomycetes species*	Inotodiol (lanostane triterpenoid)	Decreases nuclear β-catenin Inhibits TCF/β-catenin transcriptional activity	[198]
		Bioactive secondary metabolites of aromatic and heterocyclic nature		[200]
Bacteria	marine actinomycetes (*CKK1019 strain*)	chromomycins A2 and A3	-	[201]
	Marine sponge	Smenospongidine (sesquiterpenoid quinone)	Promotes the proteasomal degradation of intracellular β-catenin	[204]
Marine organisms	Marine sponge Holothuria *Peniagone sp.*	Aeroplysinin-1 (brominated tyrosine)	Promotes the proteasomal degradation of intracellular β-catenin -	[205]
		Sesterterpenoid and Steroid Metabolites		[206]
		Quinones	-	[207]
		Extract	-	[96]
	Holothuria *Molpadia musculus*	Extract	-	[96,202]
	Crustacean *Calocarides quinqueseriatus*	Extract	-	
	Crustacean *Munidopsis antonii*	Extract	-	
	Brittle star *Ophiura irrorata*	Extract	-	
	Toads	Telocinobufagin (Bufadienolides)	Phosphorylation of GSK3β	
Others	Buthus martensi	Peptide rBMK AGAP	Decreases β-catenin and p-GSK3β	[203]
